# Update on cryptogenic organizing pneumonia

**DOI:** 10.3389/fmed.2023.1146782

**Published:** 2023-04-20

**Authors:** Elzbieta Radzikowska, Justyna Fijolek

**Affiliations:** III Department of Lung Diseases and Oncology, National Tuberculosis and Lung Diseases Research Institute, Warsaw, Poland

**Keywords:** cryptogenic organizing pneumonia, secondary organizing pneumonia, acute fibrinous organizing pneumonia, cicatrices organizing pneumonia, prednisone, clarithromycin

## Abstract

Cryptogenic organizing pneumonia (COP) is a form of idiopathic interstitial pneumonia that results from the pulmonary reaction to various unidentified injuries. Secondary organizing pneumonia is diagnosed when the triggering factor has been identified; it is mainly caused by infections, toxic substance exposure, drugs, connective tissue diseases, malignancies, autoimmune diseases, bone marrow, or organ transplantation, and radiotherapy. There has been an increase in the number of reports of drug-induced organizing pneumonia (OP). New biological therapies, interferon, monoclonal antibodies, anti-interleukin antibodies, and PD1/PDL-1 inhibitors may induce this specific pulmonary reaction. The classical form of COP is usually subacute and does not manifest as severe disease. Patients maintain sufficient respiratory function, and treatment with steroids is usually effective. Several specific forms of OP (e.g., the cicatricial variant or acute fibrinous type) have distinct clinical and histological features, require higher doses of immunosuppressive drugs, and have a worse prognosis. In the era of administering steroid-sparing therapies for the treatment of interstitial lung diseases, connective tissue dases, and other conditions, it is important to emphasize this type of therapy for patients with COP.

## Definition

Organizing pneumonia (OP) is a specific pulmonary reaction to a diverse range of pneumotoxic agents, both internal and external, which produce a radiologically and histologically characteristic type of inflammatory lesion that causes the distal airways to fill with an organizing fibrous exudate and inflammatory cells, in the absence of disrupted lung architecture (1–6).

According to the classification of idiopathic interstitial pneumonias proposed by the European Respiratory Society and the American Thoracic Society, OP is a separate clinical entity within this large group of diseases (1).

OP is regarded as cryptogenic organizing pneumonia (COP) when the causative factor has not been identified, and secondary organizing pneumonia (SOP) when the possible cause of disease is known (7–18) (Tables 1–3).

**Table 1:** Autoimmunological diseases and other causes of SOP.

Autoimmunological diseases	Rheumatoid arthritis
	Dermatomyositis/ myositis
	Sjögren’s disease
	Scleroderma
	Ankylosing spondylitis
	Behcét’s disease
	Mixed cryoglobulinemia
	Periarteritis nodosa
	Systemic lupus erythematous
	Chronic thyroiditis
	Inflammatory bowel diseases
Other diseases	Acute respiratory distress syndrome (ARDS)
	Hypersensitivity pneumonitis
	Chronic eosinophilic pneumonia
	Overlap with nonspecific interstitial pneumonia (NSIP) and usual interstitial pneumonia (UIP)
	Acute exacerbation of idiopathic interstitial pneumonia (with UIP pattern)
	Cystic fibrosis
	Emphysema
	Bronchiectasis
	Choking
	Distally from a closed bronchus
	As part of a reaction around an abscess or infiltrate in the granulomatosis polyangiitis (GPA)
	Sarcoidosis
Neoplastic diseases	Tumors of the respiratory and digestive system
	Lymphoproliferative and myeloproliferative disorders
Transplantation	Bone marrow
	Parenchymal organs including lungs
Other diseases	Chronic heart or kidney failure
	Common variable immunodeficiency and other immune disorders
	Coronary by-pass grafts
Exposition	Toxic fumes (industrial gases, hydrogen sulfate)
	e-cigarette or other vaping products
	Cocaine inhalation
	Marijuana smoking

**Table 2:** Infectious factors which can induce SOP.

Viral	Human immunodeficiency virus (HIV)
	Adenoviruses
	Influenza and Parainfluenza
	SARS- CoV-2
	SARS -CoV
	MERS- CoV
Bacterial	Mycoplasma sp.
	Chlamydia sp.
	Legionella pneumophila
	Streptococcus pnumoniae
	Staphylococcus aureus
	Actinomyces israeli
	Serratia sp.
	Nocardia sp.
Fungal	Aspergillus sp.
	Pneumocystis jiroveci
	Cryptococcus neoformans
	Penicyllium sp.
Protozoan	Plasmodium vivax

**Table 3:** Drug induced SOP.

Antibiotics, antibacterial, and antifungal chemotherapeutics	Minocycline,
	Nitrofurantoin
	Cephalosporins Amphotericin
Antiarrhythmic	Amiodarone
	Beta- blockers
	Phenytoin
	Hydralazine
	Timolol
Biological agents	Interferons
	Trastuzumab
	Rituximab
	Bortezomib
	Ceritinib
	Tocilizumab
	Etanercept
	Infliximab
	Ipilimumab
	Other new biological drugs
Kinases inhibitors	Sirolimus, Everolimus
	Anti EGFR inhibitors
	Anti ALK inhibitors
PD-1/PD-L1 inhibitors	Pembrolizumab
	Atezolizumab
	Nivolumab
Antineoplastic chemotherapeutics	Azathioprine
	Chlorambucil
	Cladribine
	Bleomycin
	Busulfan
	Mitomycin
	Methotrexate
	Doxorubicin
	Daptomycin
	Oxaliplatin
	Thalidomide
Radiotherapy	Particularly breast
Other	Statins
	Dihydroergocryptine
	Penicillamine
	Propylthiouracil
Antiepileptics	Carbamazepine

For all possible causes of OP induced by treatment see www.pneumotox.com.

The disease is classified into the following forms according to the course and nature of lesions detected during histological examination: OP, acute fibrinous organizing pneumonia (AFOP), and cicatricial variant of organizing pneumonia (CIOP) (1, 5, 6, 19–25).

Focal OP is a specific form that usually progresses asymptomatically; lesion resection is sufficient treatment in many cases (3, 5, 6).

## Epidemiology

The epidemiology of OP is poorly documented. The incidence of OP differs among populations; it is estimated to be 1.97–7/100000 (3, 5, 6). An Icelandic study revealed incidences of 1.10/100000 for COP and 0.87/100000 for SOP (26). The incidence of COP has been decreasing in recent years because of improvements in the diagnosis of causative factors. Approximately 3% of patients with interstitial lung disease are presumed to have a diagnosis of OP (5, 6). According to a Greek registry, the COP prevalence is approximately 5% among patients with interstitial lung disease; a similar registry in Spain indicates that approximately 10% of patients with interstitial lung disease also have COP (26, 27). The number of patients with OP described in previous reports varies from a few cases to 48 in the study by Lazor et al., 66 patients with COP in the study by Radzikowska et al., 76 patients with COP in the study by Yoo et al., and 100 patients in the study by Yilmaz et al. (14, 28–39). Recently, Zhang et al. reported a cohort of 1,346 patients with OP, among which 176 had COP and 1,170 had SOP (40). Epler et al. evaluated 2,500 specimens from patients with interstitial lung disease; 57 (2.3%) cases exhibited bronchiolitis along with an organizing inflammatory exudate in the alveolar lumen (5).

The disease is most often diagnosed during the 5th or 6th decade of life, although cases in children have been reported. Generally, the disease exhibits no ethnicity- or sex-specific bias. Although some studies revealed that greater proportions of patients were women and nonsmokers, others indicated that OP was more common in men. At the time of diagnosis, < 30% of patients were smokers, and > 55% of patients were nonsmokers (28–40).

## Etiology and pathogenesis

Damage to the alveolar basement membrane and type II pneumocytes induced by various intrinsic and extrinsic factors is an important aspect of OP development. Vascular endothelial cells are mostly undamaged. This disruption of alveolar integrity results in the leakage of a fibrotic inflammatory exudate into the alveolar lumen, which subsequently expands into alveolar ducts and respiratory bronchioles (3, 5, 6, 41–43). Polymorphic inflammatory cells and fibroblasts migrate; undergo transformation into myofibroblasts; and bind to fibronectin, collagen I, procollagen III, tenascin C, and proteoglycans within the loose extracellular matrix. The architecture of the lung parenchyma remains unchanged. Epithelial cells proliferate, causing re-epithelialization of damaged fragments. The loose extracellular matrix containing collagen III fibers is susceptible to degradation by metalloproteases, gelatinase, and stromelysin. This process also involves increased activity among inflammatory cells, which secrete multiple cytokines that stimulate the release of enzymes capable of degrading the fibrillar exudate; the cytokines also stimulate apoptotic activity in fibroblasts (44, 45). Vasculogenesis is another mechanism involved in the disease process; it is mediated by vascular growth factors, fibroblast growth factors, and matrix metalloproteinases. Although the role of alveolar macrophages is not fully understood, cytokines released by these cells (e.g., platelet-derived growth factor-β and interleukin [IL]-8) are involved in disease progression (3, 46). Elevated levels of IL-6, IL-8, transforming growth factor-β, monocyte chemoattractant protein-1, IL-10, and IL-12 are present in serum and bronchoalveolar lavage fluid (BALF) samples from patients with OP. These observations indicate that many inflammatory cells are involved in the disease process (3, 47–49). Moreover, reduced levels of IL-6 and transforming growth factor-β have been associated with response to treatment and disease resolution (47, 49). During the resolution process, the number of inflammatory cells decreases, and fibrin deposits are removed. Concentric clusters of myofibroblasts, collagen I fibers, procollagen III, and fibronectin are formed. These clusters move into the interstitium, forming collagen foci, and the alveolar surface undergoes re-epithelialization. High apoptotic activity has been observed in fibromyxoid lesions during vascularization; this process is important during lesion healing (44, 45). Disruptions during the resolution of intra-alveolar exudate and collagen foci result in the scarring variant of OP. In COP, the inflammatory process is generally simultaneous, and the lung architecture is preserved. The presence of fibrotic lesions usually suggests overlap syndromes, such as OP with nonspecific interstitial pneumonia or with usual interstitial pneumonia (3, 25, 50).

## Symptoms

COP usually develops in a subacute manner, such that the first pseudo-flu-like symptoms precede diagnosis by 2–3 months. These symptoms generally include a subfebrile state, cough (often dry or with minimal sputum), decreased exercise tolerance, weakness, weight loss, chest pain, and night sweats. Rarely, patients report copious sputum; even more rarely, they report hemoptysis (< 5%) or pneumothorax. Uncharacteristic symptoms can lead to a diagnostic delay of 1–5 months (28–39).

Rarely, OP progresses to a severe form with features of respiratory failure. Its main histological counterpart is AFOP (19–24), which is characterized by richly fibrinous inflammatory exudate in the alveoli, respiratory tract, and bronchioles; this exudate comprises spherical conglomerates, observed as diffuse bilateral shadows on radiographic imaging. AFOP is most often secondary to lung transplantation, allergic alveolitis, reactions to pneumotoxic agents, connective tissue diseases, and infections. AFOP has a high mortality rate, particularly in transplant patients (3, 6, 28–40).

Patients with focal OP are usually asymptomatic.

Physical examinations reveal weakened protrusions in areas of lesions, along with the sound of fine-bubble rales. No lesions are detected during physical examinations in approximately 30% of patients (3, 5, 6, 28–39).

COP patients usually do not show clubbed fingers, but this phenomenon is evident in patients with overlap syndromes such as usual interstitial pneumonia or nonspecific interstitial pneumonia; it may also be present in patients with underlying diseases (f.c. circulatory insufficiency, neoplastic) (3, 5, 28–39).

## Laboratory tests

Laboratory tests detect increases in serum C-reactive protein concentration, erythrocyte sedimentation rate, and lymphocytosis in approximately 40% of patients.

OP may precede the onset of autoimmune disease by many months or years. Thus, appropriate serological diagnostics should be conducted to assess rheumatic factor, anti-cyclic citrullinated peptide antibody, anti-nuclear antibody, anti-topoisomerase antibody (anti-SCl-70), anti-Jo-1 antibody, anti-Ro52 antibody, anti-dsDNA antibody, and other such factors (3, 5, 6, 28–39).

## Radiological examination

### Chest radiography

**Figure 1:**
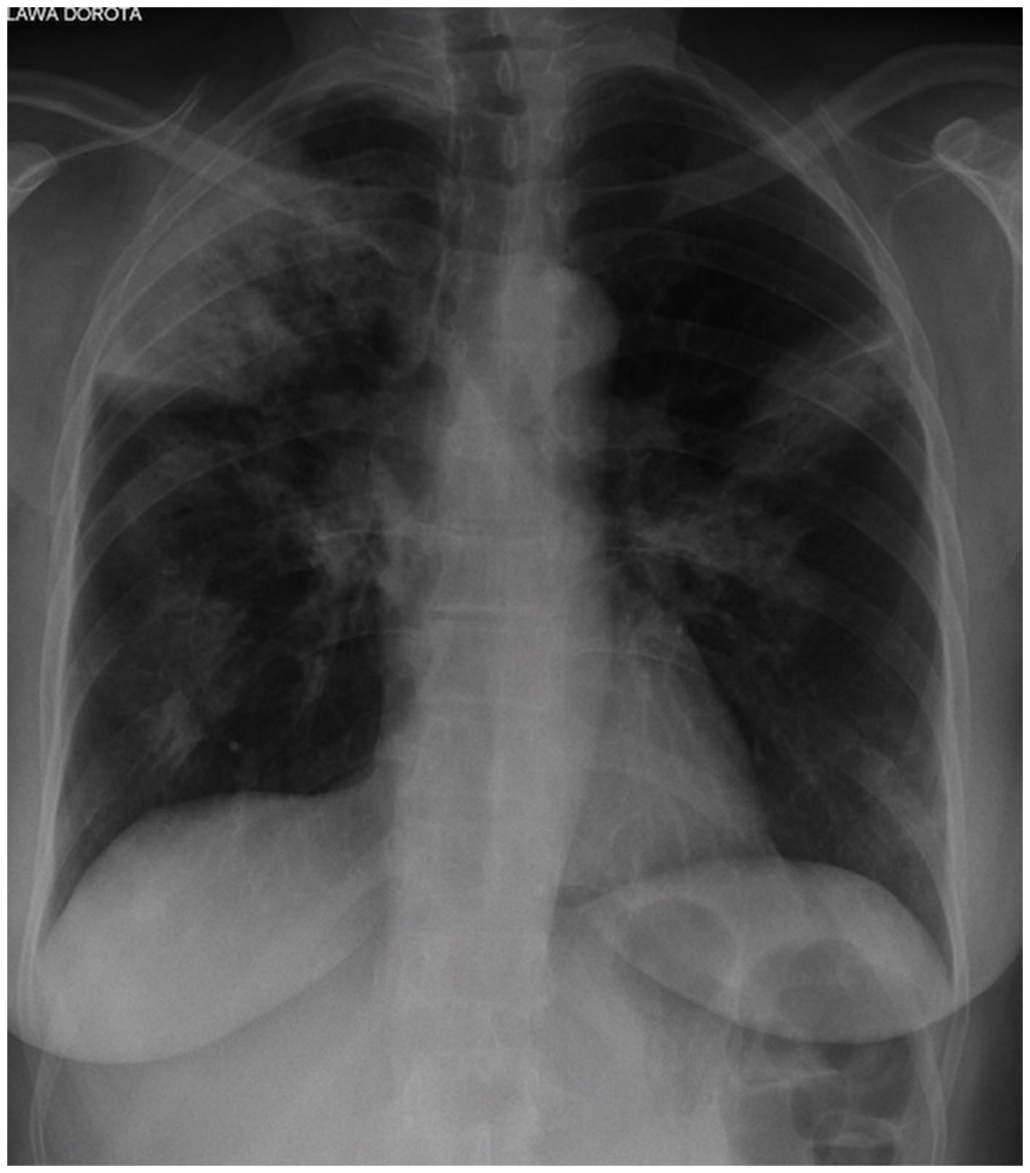
Chest X-ray of OP. Bilateral opacities localized in the peripheral parts of the both lungs.

Routine chest radiography of patients with OP usually reveals bilateral opacities localized in peripheral parts of the lungs, without abnormal lung volume (Figure 1). Less frequently, diffuse lesions may be present, in the form of fine-spotted and nodules, single nodules, or mass like lesions. The changes are usually localized in the middle and lower lung fields, although they are also observed in the upper fields in one-third of patients. Lesions undergo migration in 50–75% of cases, and approximately 10% of patients exhibit spontaneous regression (3, 5, 6, 28–41, 43). The standard radiography is poorly sensitive and specific for OP.

### Chest computed tomography

**Figure 2:**
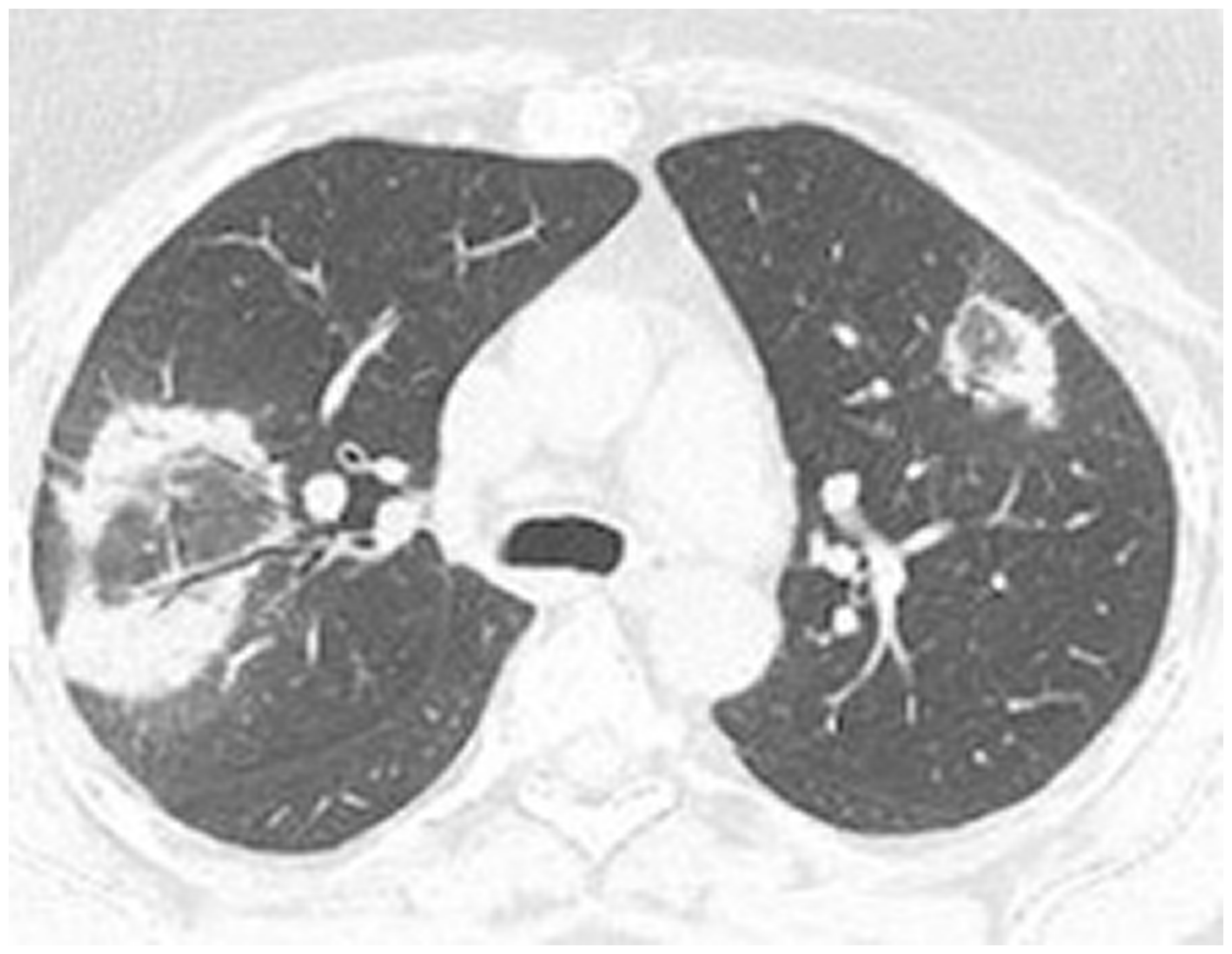
High resolution computed tomography scan of a patient with COP. Bilateral ground glass opacities with associated peripheral consolidations forming an atoll sign.

**Figure 3:**
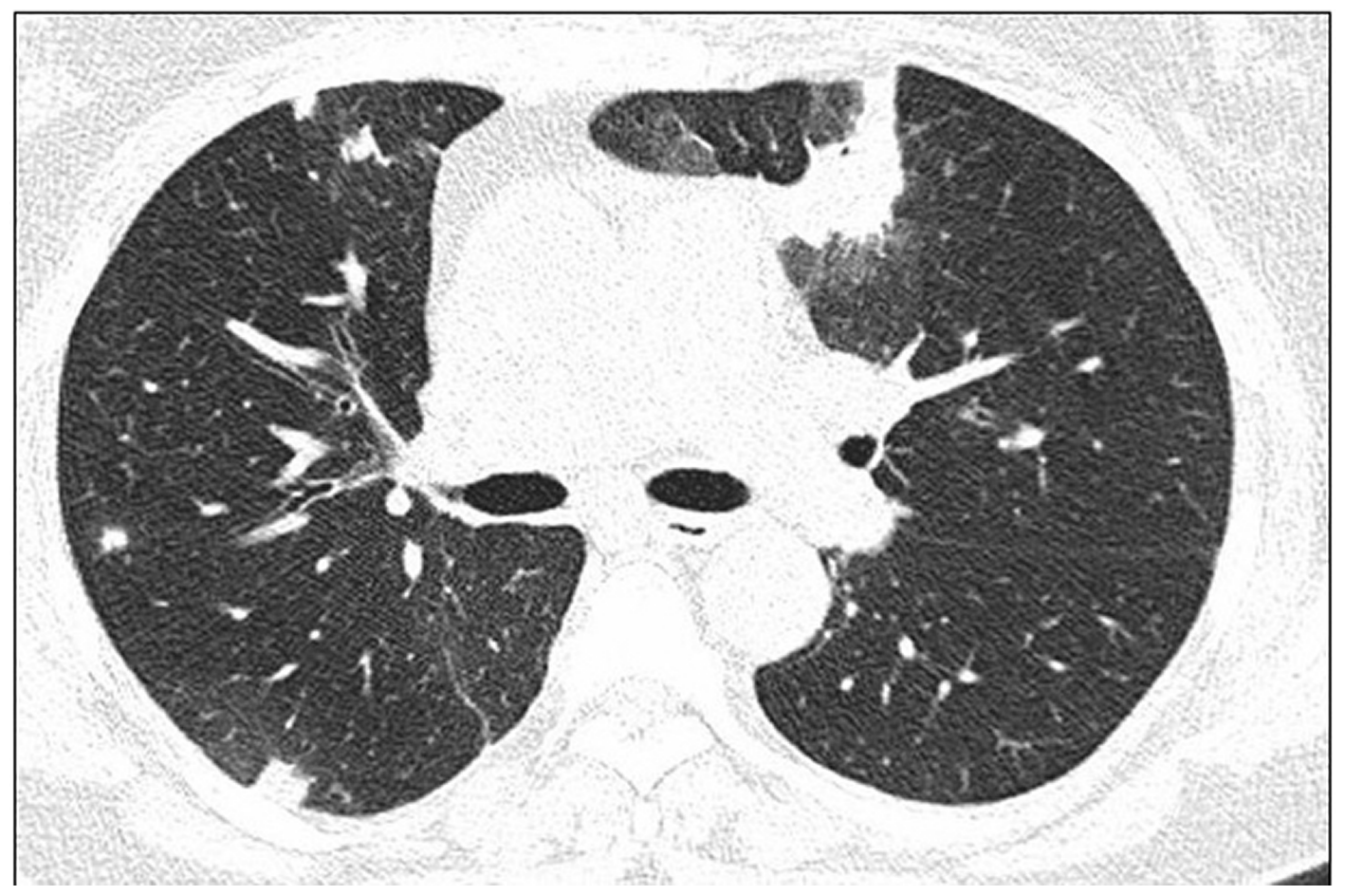
High resolution computed tomography scan of a patient with COP. Bilateral nodular consolidations.

**Figure 4:**
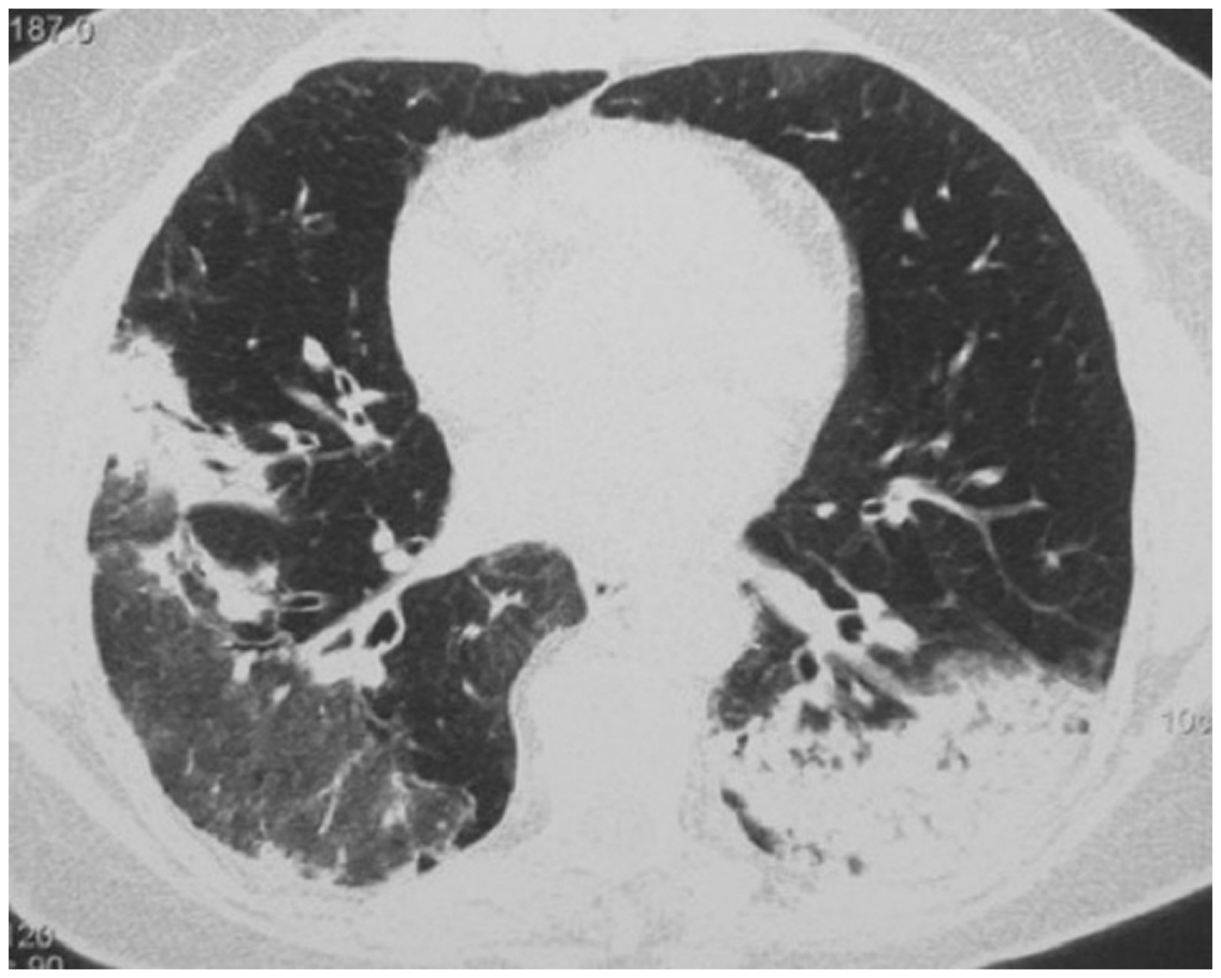
High resolution computed tomography scan of a patient with COP. Massive bilateral opacities with presence of air bronchogram and accompanying ground glass opacities on the right lung.

**Figure 5:**
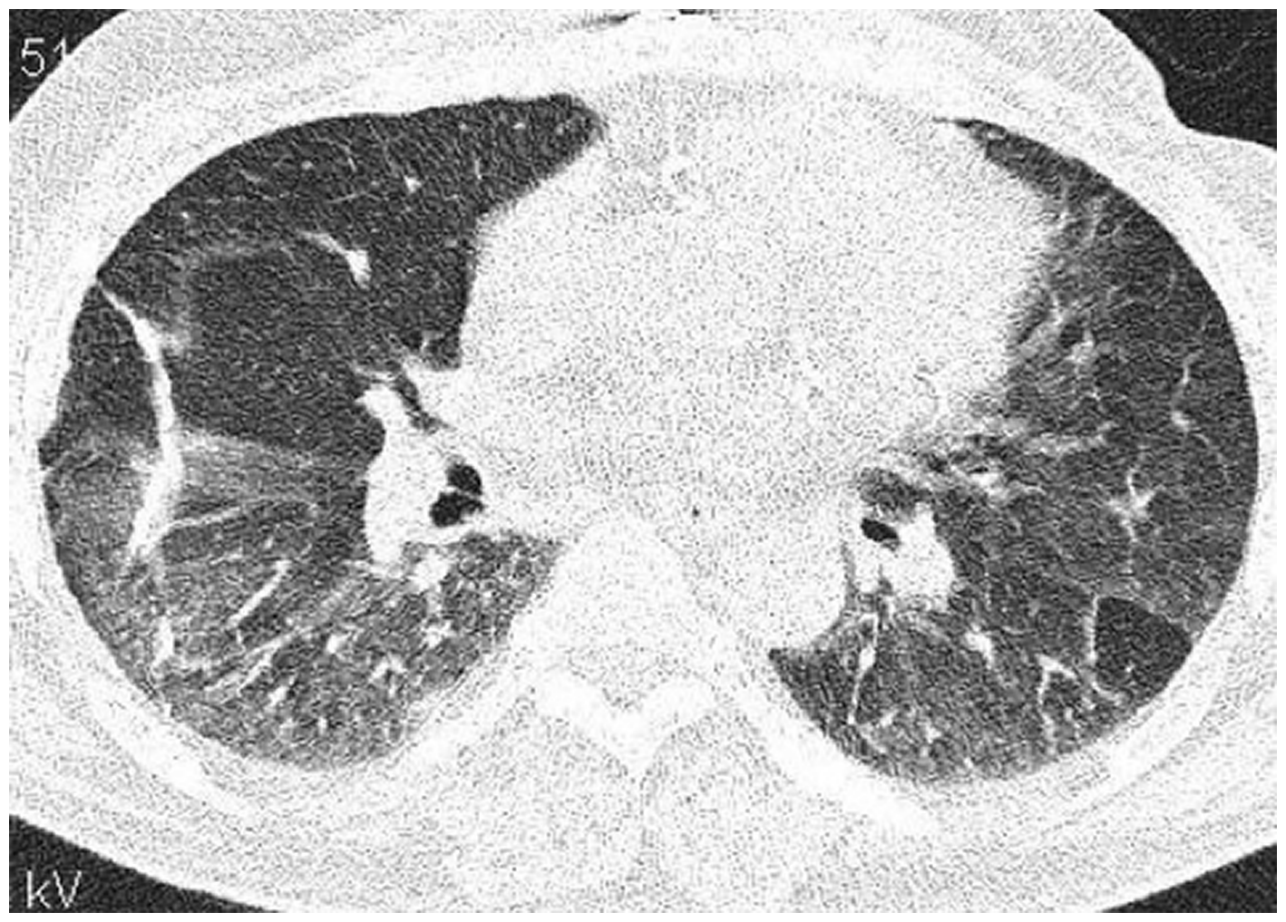
High resolution computed tomography scan of a patient with SOP in the course of dermatomyositis. In both lungs evidence of GGO with patchy distribution and parenchymal banding with “arcade” sign typical of OP. Status post lung biopsy on the right side.

**Figure 6:**
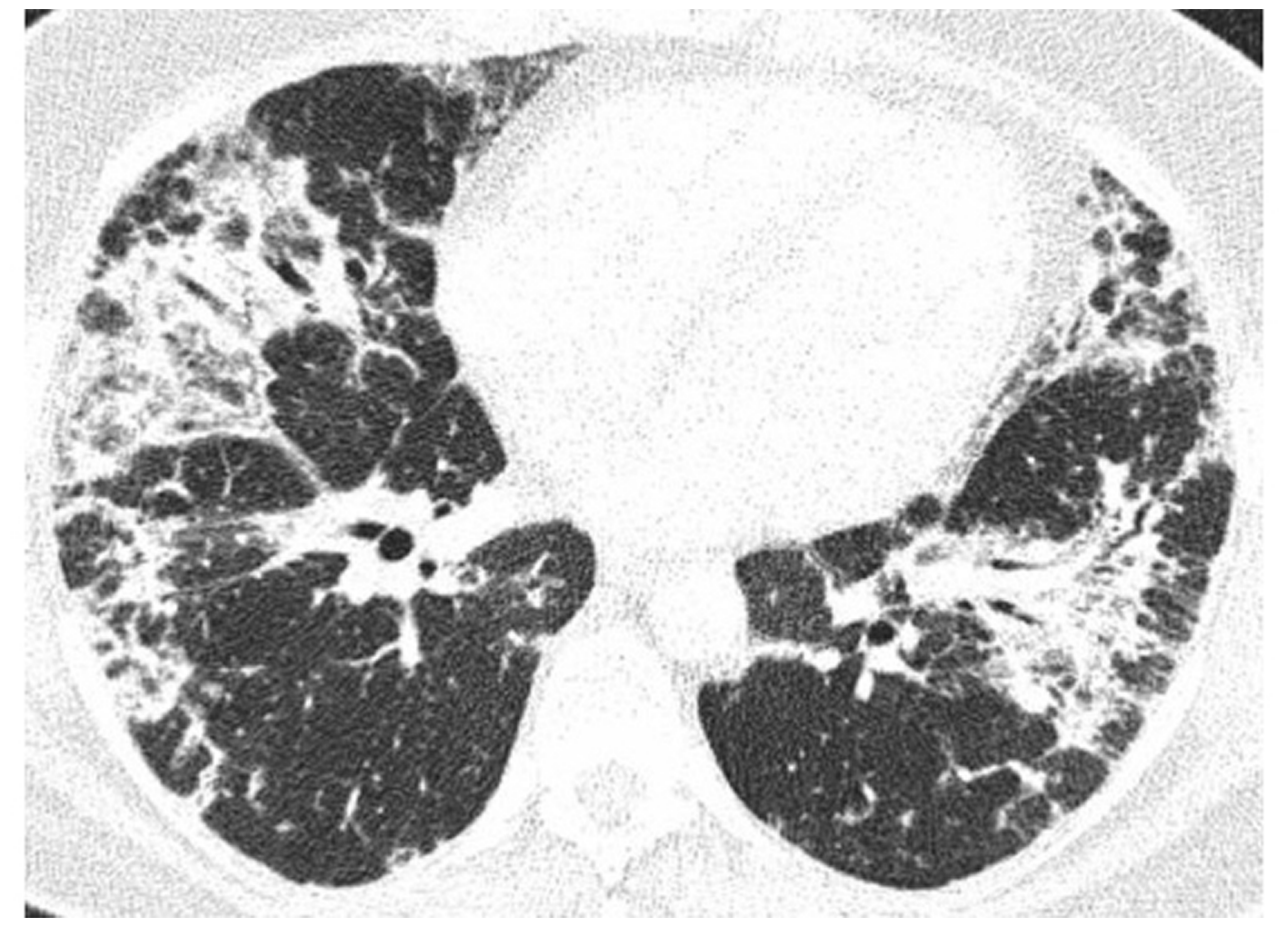
HRCT scan. Etanercept induced AFOP, in patient with rheumatic arthritis and pulmonary fibrosis. Bilateral consolidations with air bronchogram, interlobular septa thickening, and ground glass opacities predominated in the peripheral parts of both lungs.

High-resolution computed tomography (HRCT) is the gold standard in the evaluation of OP. It reveals multifocal areas of consolidation, often with a characteristic air bronchogram. Additionally, patchy alveolar consolidations, nodules, areas of ground glass opacity, perilobular infiltrations, bronchial wall thickening, and reticular fibrous changes may be present in peripheral parts of both lungs. Thickening around areas of ground glass opacity with an “atoll” or “crazy-paving” pattern may also be present, although it is less common. Additionally, nodular lesions, pleural thickening, and rarely enlargement of hilar and mediastinal lymph nodes, are present; emphysema or pleural effusion may also be observed (28–40). Honeycomb-type lesions are not in the spectrum of pulmonary changes observed in COP but might be evident in patients who exhibit interstitial pulmonary fibrosis with a component of OP (Figures 2–6).

The micronodular form of OP is more frequently observed during the course of OP accompanying myeloproliferative diseases or infections, or after exposure to marijuana smoke (3, 28–40).

Some case series have reported higher numbers of solitary mass-like lesions mimicking lung neoplasia, whereas other case series have indicated that such lesions were present in only a few patients (Table 4) (3, 6, 40, 51, 52).

**Table 4:** Radiological findings detected in OP patients.

The most frequent	Patchy alveolar opacities
	Consolidations with and without air bronchogram
	Migration of lesions
	Subpleural localization
	Bilateral lesions
Frequent	Ground glass opacities
	Reversed halo sign
	Nodules
	Multiple masses
	Septal thickening
Rare	Lymphadenopathy
	Reticular opacities
	Centrilobular nodules
	Perilobular and linear opacities
Extremely rare	Diffuse micronodules
	Pneumothorax
	Pleural effusion

## Bronchoscopy and bronchoalveolar lavage

Bronchoscopy and bronchoalveolar lavage are used to exclude possible causes of the observed lesions (i.e., infections, neoplasia, eosinophilic pneumonia, and alveolar hemorrhage) (53). The lymphocyte percentage in BALF is usually high (20–40%), and eosinophils and neutrophils are present (approximately 7–10%). In approximately 40% of patients, lymphocytosis with a decreased CD4^+^/CD8^+^ lymphocyte ratio is detected, but this is not a pathognomonic finding for the disease. Patients with an increased eosinophil percentage may have overlap syndrome comprising OP with chronic eosinophilic pneumonia.

Specimens obtained *via* transbronchial lung biopsy may be insufficient for diagnosis, particularly when the clinical and radiological pictures are questionable. The positive and negative predictive values of transbronchial lung biopsy were approximately 94 and 40%, respectively (54). Cryobiopsy may be an effective diagnostic route (55, 56). However, the gold standard for diagnosis is histological evaluation of specimens obtained by open lung biopsy (1, 3, 5, 6, 57–59).

## Histological examination

**Figure 7:**
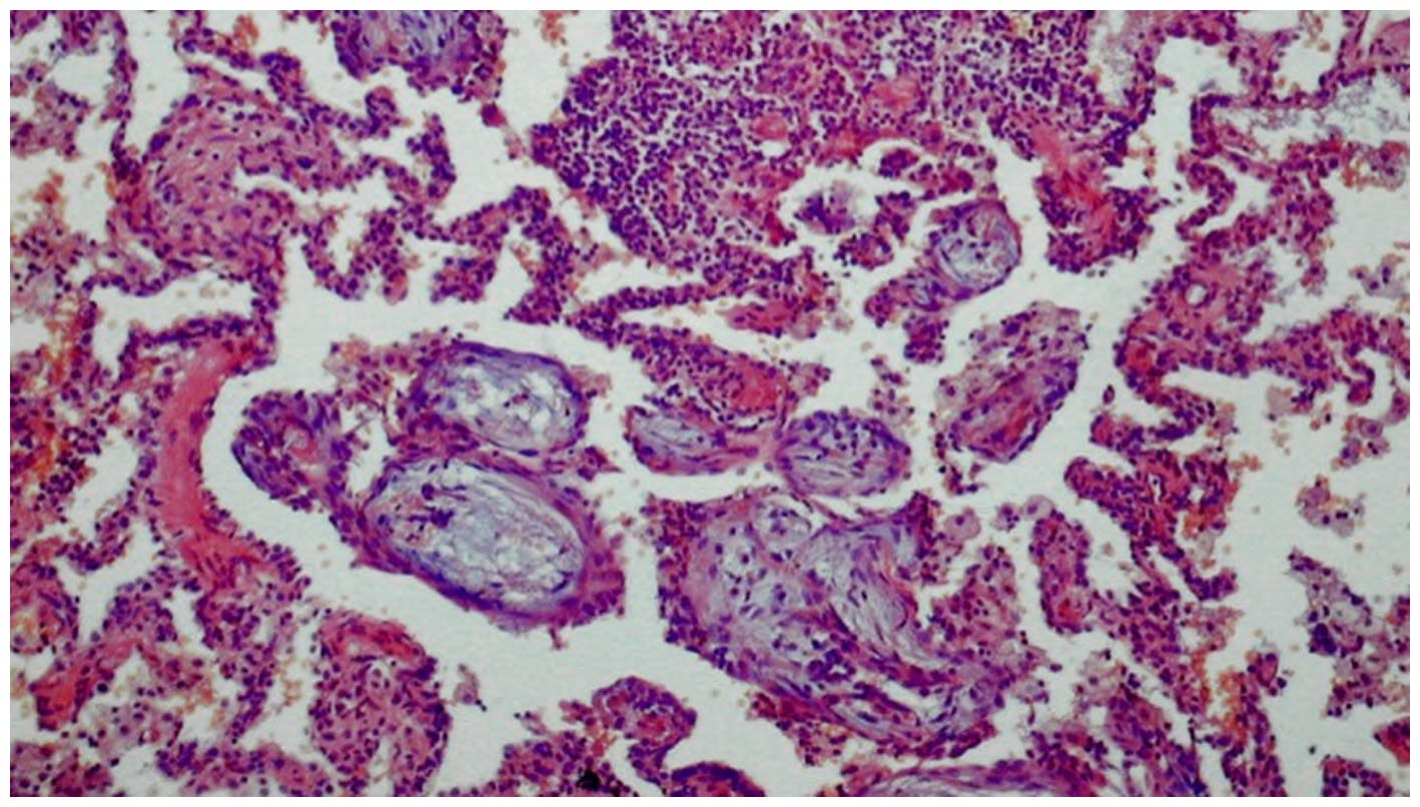
Histological image of organizing pneumonia (H-E). Alveoli filled with loose tissue forming polypoid formations extending into the respiratory tracts with a small diffuse inflammatory infiltrate in the interalveolar spaces.

In OP, histological examination detects a rich fibrous exudate in alveolar spaces, which expands into alveolar ducts and respiratory bronchioles; it forms characteristic polypoid lesions accompanied by a polymorphic inflammatory infiltrate composed of macrophages, plasma cells, lymphocytes, and eosinophils (Figure 7).

Some degree of inflammation is observed in the lung interstitium, but the lung architecture remains intact. The lesions usually have a unilocular appearance; typically, advanced fibrotic changes are not present at the time of diagnosis. OP involves extensive apoptotic activity in the extracellular matrix. The damaged lung is remodeled by re-epithelialization and repair of the basement membrane, along with resorption of the accompanying inflammatory exudate.

During the development of CIOP, clusters of fibrin and banded fibrous lesions are present in peribronchial spaces, sometimes accompanied by foci of dendritic ossification. This form of disease presumably results in a predisposition toward the onset of nonspecific interstitial pneumonia. Rarely, typical lesions of OP are accompanied by poorly formed granulomas (3, 5, 41).

Similarly, AFOP is detected in cases of sporadic disease with a severe and aggressive course. Histological criteria for this form are the presence of balls of organized fibrin conglomerates in the alveoli and respiratory ducts, patchy distribution in the absence of vitreous membranes, inflammatory granulomas, eosinophilic pneumonia, and bronchiolitis with abscess formation (41, 42, 57).

## Pulmonary function tests

Despite the extensive radiographic changes, ventilatory parameters usually are not significantly impaired; they are within normal ranges in approximately 30% of patients. Impaired transfer rate for carbon monoxide and restrictive-type ventilatory abnormalities are most commonly detected in approximately 60–70% of patients. Features of hypoxemic respiratory failure are less frequently observed. In contrast, increased ventilatory and blood gas abnormalities are more often associated with SOP (28–38, 60).

Arterial hypoxemia at rest is uncommon (< 20% of patients), whereas arterial hypoxemia during exertion is more frequent and corresponds to impairments in diffusion capacity for carbon monoxide (3, 6, 28–40).

## Diagnosis

The diagnosis of the disease is based on:

- Analysis of the clinical and radiological findings
- Exclusion of all possible causes of OP
- Histological examination of samples obtained by open lung biopsy or transbronchial lung biopsy
- Examination of lung samples obtained during transbronchial lung biopsy may be insufficient for patients with atypical clinical and radiological pictures; the OP lesions may be accompanied by other forms of interstitial pulmonary fibrosis, infections, and tumors

## Diagnosis of AFOP

- Rapid clinical course leading to respiratory failure
- Radiological picture of extensive interstitial opacities
- Histological findings of richly fibrinous inflammatory exudate within the alveoli and in the respiratory tract, along with the formation of fibrin conglomerates, while excluding the presence of vitreous membranes, eosinophilia, bronchiolitis, and granulomas

Generally, a diagnosis of COP should be based on multidisciplinary consultation (1, 3, 6).

## Differentiation

Diagnosis requires a specific determination of possible etiology, particularly with respect to potentially reversible causes of disease, such as infections (e.g., coronavirus disease 2019), autoimmune disease, neoplastic disease, drug reactions, and toxic lung damage. Additionally, there is a need to determine whether OP is a component of another disease, including nonspecific interstitial pneumonia or usual interstitial pneumonia.

Chronic eosinophilic pneumonia may resemble OP in terms of clinical and radiological pictures. The disease more often affects women in the 5th to 6th decades of life, usually with a history of allergy, as well as sputum and > 25% eosinophils in BALF.

Opacities with air bronchogram are visualized by computed tomography, although they frequently do not exhibit migration; these shadows can be a sign of mucosa-associated lymphoid tissue lymphoma or adenocarcinoma of the lung.

Other forms of idiopathic interstitial pneumonitis should be considered in the differential diagnosis, and the key assessment comprises evaluation of lung sections.

Nodular lesions that raise suspicion of malignancy require histological verification. Patients who have undergone radiation therapy for breast cancer are at high risk, such that 1–3% subsequently develop OP (3, 6, 59, 61–65).

## Treatment

Treatment strategies for COP should be based on assessment of the patient’s clinical status and the disease severity, with particular attention to respiratory sufficiency. The disease spontaneously resolves in <10% of cases.

Patients with localized COP treated by surgical resection of the lesion have a good prognosis; this is frequently sufficient treatment (40).

Steroid administration is the standard treatment for COP and SOP. Clinical and radiological improvements occur within a few days after the initiation of treatment; however, approximately 50% of patients experience recurrence, including approximately 20% who have multiple recurrences of COP. Relapses mainly occur during the first year after the initial episode. New infiltration occurs during steroid tapering (< 10 mg of prednisone) or within a few months after the completion of treatment. The factors associated with greater probability of relapse have not been fully elucidated, and different case series have yielded discordant results. However, putative factors include diagnostic and treatment delays, respiratory insufficiency, extension of pulmonary infiltrates, the presence of multifocal opacities, higher levels of inflammatory markers, and the presence of gastroesophageal reflux. Recurrence does not affect the mortality rate, and prolonged steroid treatment does not prevent recurrence. However, prolonged steroid treatment increases the likelihood of adverse events during therapy, which occur in 12–50% of patients; some events can be very severe, including pulmonary embolism, vertebral fracture, diabetes, and tuberculosis. The possibility of secondary causes of disease (e.g., autoimmune diseases and malignancy) should be considered in patients with recurrent OP. The optimal steroid dosage regimen and duration of therapy have not been established. The most common recommendation comprises the administration of 0.5–1 mg/kg per day of prednisone in gradually decreasing doses for 6–12 months. The dose and duration of treatment should be modified in accordance with the patient’s clinical condition. Relapses are usually treated with lower doses of steroids (approximately 20 mg/day for 6 months) (28–40) (Table 5).

**Table 5:** COP treatment with corticosteroids – observational retrospective studies of cohorts over than 20 patients (COP patients were extracted from presented populations).

Authors	Patients no	Initial dose of PRE	Duration of treatment (months)	Complete response rate *n* (%)	Rapid progression	Relapse *n* (%)	Adverse events	Observation period (months)
Epler et al. (5)	37	NR	3–12	24 (65%) 9 (%) partial response		8 from 24 (33%)	2 deaths from OP 2 pulmonary embolism 1 death aneurysm	Mean 4 years, range 2–10 years
King (6)	96	NR GR:1–1.5 mg/kg/d	NR GR: About 12	76 (63%)		NR 30 (37%) persistent disease	11 (12%) patients died	NR
Lohr et al. (62)	20	51 mg/d	Mean 12.7			4(13%)	5-year survival 73%	36–48
Cazzato et al. (33)	43	40 mg/d (20-120 mg/d)	6–9	NR 5 spontaneous remissions	2	15 (26%)	3 deaths	NR
Lazor et al. (28)	48	50 ± 17 mg/d	>12 68% patients were still on treatment	20 (42%)		28 (58%)	12(25%) No deaths	35 ± 31 23 median
Barroso et al. (63)	33	56 ± 12 mg/d	Non relapsed 12 ± 3; Relapsed 42 ± 10, *p* < 0.01, multiple relapsed 98 ± 48 months, *p* = 0.03	14/32 (44%)		18/32 (56%)	2 patients died (one of tuberculosis)	33 patients alive at 144 months 2 patients were on steroids in the time of analysis
Yoo et al. (14)	76 36- PRE 40- PRE+ CTX	54.6 ± 12.6 mg/d	10.5 (4.0–14.7)	35 (46%)	Rapid progression 5 (6.6%) Stabile 2 (2.6%)	14 (20%)	11 (14.5%) Disease related death	Median 38.2 (13.0–68.6)
Sveinsson et al. (64)	40	42.4 mg/d	NR	NR		8 (20%)	NR	Mean 4.7 years
Drakopanagiotakis et al. (38)	30	NR	Nr	17 (47%)		13 (43%)	2 (5.3%) 1-year mortality	NR
Onishi et al. (39)	40	0.5–0.8 mg/kg/d	1–6	25 (62%)		15 (38%)	No deaths caused by OP NR	NR
Radzikowska et al. (29)	22 (respiratory sufficient)	0.67 ± 0.24 mg/kg/d	mean of 8.59 ± 3.05	21 (100%)		12 (54.5)	8 (6.5%) patients (1 death)	67 ± 45.6
Saito et al. (66)	33	11 severe patients received 1.0 methylprednisolone iv. for 3 days	Meantime 282 ± 183 days in non-relapse group and 281 ± 174 days in relapse group	23 (70%)		10 (30%)	NR	NR
Zhou et al. (37)	73	20-200 mg/d	6–12 months	60 (68.5%)		23 (31.5%)		50.3 ± 26.8 months (range 9.3–96.4 months)
Zhang et al. (40)	53	1–2 mg/kg/d	Min. 12	12 (23%)	PR7 (13%)	35 (70%)	3 deaths	98.3% 5-year survival
Radzikowska unpublished data	32	0.73 ± 0.24 mg/kg/d	Mean 8.68 ± 4.08	32 (100%)		17 (53%) multiple relapse 15(%)	17 (53%) 2 deaths	64.3 ± 46.0 No one on steroids in the time of analysis

CTX, cyclophosphamide; GR, general recommendations; NR, not reported; PRE, prednisone; PR, partial response.

Clinically severe forms of the disease, which mainly occur in patients with SOP, have been treated with intravenous boluses of corticosteroids (500–1,000 mg methylprednisolone intravenously for 3–5 days with subsequent oral prednisone treatment at 1 mg/kg) and other immunosuppressive drugs (e.g., cyclophosphamide, azathioprine, cyclosporine A, mycophenolate mofetil, and rituximab), usually in combination with glucocorticoids (3, 6, 24, 38–40).

Generally, COP exhibits a very good response to corticosteroid treatment in patients with respiratory sufficiency, good clinical status, and no symptoms of severe disease; steroid-sparing therapies with macrolides have demonstrated promising results. There is increasing evidence regarding the efficacy of clarithromycin (CAM) treatment in COP patients without features of respiratory distress (67–75) (Table 6). The most effective dosage regimen is oral administration of CAM at 2 × 500 mg/day for 3 months. Significant improvement occurs after 1 month of treatment, rather than within a few days (e.g., the duration expected during steroid treatment). More than 80% of patients achieve complete regression of lesions after a 3-month period of treatment, and the recurrence rate is <10%. Adverse effects of this treatment are limited to allergic reactions and dyspeptic symptoms (68). Lower doses of CAM (2 × 500 mg for 1 week, 500 mg for 3 weeks, and 250 mg for 8 weeks) in combination with prednisone also led to good therapeutic effects (63% complete regression and 38% partial regression), but 80% of the patients exhibited OP recurrence. In the control group treated for 6 months with prednisone alone, the proportions of patients who achieved complete and partial remission were 81 and 14%, respectively; relapses occurred in more than half of the patients (75).

**Table 6:** COP treatment with clarithromycin – observational retrospective studies.

Author	Patients no	Treatment	Dose of CAM	Initial dose of PRE	Duration of treatment (months)	Complete response rate n (%)	Partial response > 50% n (%)	Relapse *n* (%)	Adverse events
Ichikawa et al. (67)	6	Erythromycin 600 mg/d			3–4	6 (100%)		NR Observation mean 3.8 months	without
Epler et al. (5)	37	PRE		1 mg/kg/d	NR	24 (65%)	13 (26%)	33% treated with PRE	2 deaths of OP
	7	Tetracycline. erythromycin		NR	NR	3(43%)		NR	NR
	4	No treatment				2 (50%)			
Stover et al. (69)	6 (3 COP 3 ROP)	CAM	2×250 2×500		1.5–6	5 (83%)	1 (17%)	1 (17%) patient with ROP *	NR
Sveinsson et al. (64)	40	PRE	NR	Mean 42.4	NR	NR	NR	22 (55%)	1 death steroid responsive 1 death acute OP
	1	Erythromycin	NR					0	
	17	Surgery						0	
Drakopanagiotakis et al. (38)	30	PRE		NR	NR	NR	NR	38	9.4%
	3	Macrolides	NR					NR	
	9	No treatment							
Pathak et al. (71)	3 cases second line treatment 1 case first line	CAM	Two patients 2×0,5 Two patients 2×0.25	NR	5 or NER	4 (100%)		NR	NR
Radzikowska et al. (29)	40	CAM	2 ×0.5		3	35 (88%)	4 (10%)	10	1 (2.5%)
	22	PRE		Mean 0.67 ± 0.24 mg/kg/d	8.59 ± 3.05	22 (100%)	0	54	8 (36%) 1(5%) death
Petitpierre et al. (75)	16	CAM + PRE	2×0.5 for 1 week 0.5 for 3 weeks 0.25 for 8 weeks	0.75	3	10 (63%)	6 (38%)	81	0
	21 Previously published	PRE		0.75	6	17 (81%)	3 (14%)	52	0
Ciftci et al. (74)	7	CAM	2×0.5		3–9	7 (100%)		0	
Zhou et al. (37)	73	PRE		0.75	12	NR	NR	32	NR
	8	CAM		NR	4.2 ± 2.7	7 (64%)	NR	36	NR
	3	Azithromycin		NR					

CAM, clarithromycin; PRE, prednisone; COP, cryptogenic organizing pneumonia; SOP, secondary organizing pneumonia: ROP, radiotherapy induced organizing pneumonia; NR, not reported, NER -not exactly reported patient with large cell lymphoma.

CAM was effective treatment for patients with radiotherapy-induced OP.

Similar to the first-line treatment, steroids are used as treatment for relapses. However, relapses can be treated with lower doses of glucocorticoids (< 20 mg/day) or CAM (5, 68).

Prophylactic treatment of *Pneumocystis jiroveci* infection is recommended for patients receiving steroids.

There have been no randomized controlled trials of steroid or CAM treatments for COP. The recommendations for treatment are based on individual experience and the results of uncontrolled studies. Considering the treatment duration, results, adverse events, and probability of relapse, CAM should be the first choice for treatment in patients with COP who exhibit respiratory sufficiency. Steroids should be considered for patients with a severe and aggressive disease course, particularly patients with underlying conditions (e.g., connective tissue disease, malignancy, or suspected drug reaction). Among patients who fail to respond to steroids, more aggressive treatment with cyclophosphamide, azathioprine, or rituximab is recommended.

## Monitoring

Relapses of OP are common, particularly in steroid-treated patients; they most frequently occur during the period of prednisone dose reduction to approximately 5–10 mg or within 2–3 months after the completion of treatment. Therefore, follow-up examinations are recommended during the first year after completion of treatment. Additionally, particular attention is necessary for patients whose lesions have not completely resolved, and for patients in whom OP is regarded as the first symptom of a developing connective tissue disease. Factors associated with a higher risk of recurrence are diagnostic delay (> 2 months), severe disease, the presence of multifocal lesions and dilatation with stretching on radiographs, transfer factor of the lung for carbon monoxide <50%, hypoxemia <70 mmHg, and the presence of fibrin conglomerates and cicatricial lesions on histological examination (3, 28–40, 66, 68, 75).

## Prognosis

Typical COP has a good prognosis. Spontaneous regression occurs in approximately 10% of cases, and > 75% of patients recover fully.

Poor response to treatment occurs in patients with a disease pattern that is suggestive of OP overlap with nonspecific interstitial pneumonia, patients in whom the disease is caused by exposure to pneumotoxic agents, patients with connective tissue diseases (including diseases induced by biological drugs), patients with myeloproliferative diseases, and patients who underwent lung or bone marrow transplantation. Very rarely, COP can result in death (3, 6, 28–40, 68–75).

## Prevention

Because the cause of disease is not fully understood, there are no established methods for prevention.

Avoidance of infection is recommended; this includes efforts to undergo immunization against influenza, pneumonia, and coronavirus disease 2019.

## Variants of organizing pneumonia

### AFOP

More than 150 patients with AFOP have been described thus far, and the significance of the histological findings remains unclear (24, 76–80). This disease may be idiopathic or (more frequently) secondary, related to diffuse alveolar damage, adverse drug reaction, transplantation, and connective tissue disease. Although AFOP was first identified in patients with acute respiratory failure, the course of the disease can be acute or subacute. Confluent, bilateral, massive consolidations in basal areas of the lungs are usually observed. Patients with a fulminant clinical course and rapid progression to death exhibit radiological findings similar to diffuse alveolar damage, along with diffuse consolidations and ground glass opacities in lower parts of the lungs. In patients with a subacute clinical course, the radiological picture is more compatible with typical OP, including diffuse and focal consolidations with an air bronchogram and ground glass opacities. These patients also have better prognoses. Histologically, AFOP is characterized by the presence of organized fibrinous balls filling the alveoli, combined with type II pneumocyte hyperplasia and the absence of hyaline membrane. In the original report by Beasley et al., 17 patients with AFOP were identified among 114 cases with diffuse alveolar damage and OP (19). Relevant possible causes of the lesions were present in 11 patients; the possible cause of disease was unknown in the remaining 6 patients. The prognosis of AFOP is poor; 50% of the patients in the cohort presented by Beasley et al. died, including all patients who received mechanical ventilation. Onishi et al. identified 19 cases of idiopathic AFOP in a group of 34 patients diagnosed with AFOP. Corticosteroids were effective in 94% of the patients in the AFOP group, but relapses occurred in 76% of the patients (23). Additionally, a higher corticosteroid dose was needed during recurrence than during the initial course of disease. These patients had favorable outcomes, but two deaths from respiratory failure occurred among patients with underlying diseases. In that study, AFOP was more common than previously reported, and the patient prognosis was better than in other studies. In a review of cases and cohorts of patients with AFOP, Chen et al. reported that 33% of patients had idiopathic AFOP; underlying conditions responsible for disease were identified in the remaining patients (i.e., those patients had secondary AFOP) (24). Dyspnea (72%), nonproductive cough (71%), and fever (43%) were the most common symptoms, and they usually exhibited subacute onset (41%). Acute disease onset was identified in 27% of patients; it was nonspecific in the remaining patients. Bilateral consolidations, mainly in the lower and peripheral parts of both lungs, were present in 77% of patients. Consolidations, ground glass opacities, and nodules were evident in 54, 42, and 20% of patients, respectively. Lesions in the form of consolidations were more common in patients with idiopathic AFOP than in patients with secondary AFOP (70% vs. 47%, respectively). Rarely, pleural fluid (5%) and solitary nodules (1.3%) were detected in patients with secondary AFOP. Steroid administration was the most frequently prescribed therapy (88%); the dose and duration considerably varied. Immunosuppressive agents (e.g., cyclophosphamide, mycophenolate mofetil, tacrolimus, and azathioprine) were administered to 17 patients, most of whom exhibited secondary AFOP during the course of autoimmune disease. Drug-induced AFOP was identified in 17 (11%) patients; withdrawal of the possible causative drug and administration of corticosteroids were recommended (3, 6, 12).

Disease-related deaths were reported in 20% of idiopathic AFOP patients and 49% of secondary AFOP patients (80).

Moreover, close monitoring of patients with cryptogenic AFOP is recommended because it can reveal connective tissue disease or malignancy as the cause of the lesions.

### Cicatricial variant of organizing pneumonia

CIOP is a rare histological variant of OP, which has been referred to by various names, including fibrosing OP, collagenized OP, and scarring variant of OP; these names should be consolidated to a single term (16, 25, 81–83). Yousem et al. found this variant in histological specimens from 12 of 223 patients with OP, which had been collected over a 20-year period. Additionally, 30 other patients had CIOP secondary to connective tissue diseases and malignancies. In this group, the disease was mainly present in middle-aged men; bilateral nodular or reticulonodular lesions were the most frequent manifestation. Histological examination revealed that fibromyxoid material filled distant airways and alveoli; dense eosinophilic fibrosis was evident in the center of the lesions, along with preservation of the lung architecture. In the central parts of these changes, elastic tissue was revealed by specific stains (e.g., Elastica van Gieson). Mild lymphocytic bronchiolitis and patchy alveolar septal infiltrates were present. In some cases, dendriform ossifications, fibrous pleuritis, and dust deposits were present. The lesions were persistent or progressive in >50% of patients. There has been discussion of separating this variant from classical COP (25). Woge et al. reported the presence of OP in 56 cases diagnosed *via* surgical biopsy specimens that had been collected over a period of 9 years. Thirty-two of 56 cases (57.1%) exhibited ≥10% cicatricial elements within fibromyxoid balls; cicatricial elements comprised ≥50% of OP in 9 of these cases. Intraluminal ossification was present in five of these nine cases. In 6 of these patients with major cicatricial changes, the clinical condition was good over a median follow-up period of 47 months (83).

Recently, Zaizen et al. analyzed the histological findings of 121 patients with fibrotic interstitial pneumonia; they found that CIOP coexisted with usual interstitial pneumonia, nonspecific interstitial pneumonia, and chronic hypersensitivity pneumonia patterns. CIOP as a predominant type of lesion was present in 7 of 48 patients who exhibited any CIOP changes. CIOP was regarded as a variant of OP, as well as a histological lesion that can occur within other types of fibrotic interstitial pneumonia. Moreover, the presence of CIOP in patients with fibrotic interstitial pneumonia was associated with a low risk of acute exacerbation, slight improvement of ventilatory impairment, and better prognosis (82).

## Conclusion

Cryptogenic organizing pneumonia (COP) is a result on the pulmonary reaction to various unidentified injuries. It has usually subacute course, and rarely manifests as severe disease.

The diagnosis of COP requires detailed evaluation of possible causes of secondary disease such as: infections, toxic substance exposure, drugs, connective tissue diseases, malignancies, autoimmune diseases, bone marrow or organ transplantation, and radiotherapy.

There have been no randomized controlled trials of steroid, clarithromycin, and other immunosuppressive treatments for COP. The recommendations for treatment are based on individual experience and the results of uncontrolled studies. Considering the treatment duration, results, adverse events, and probability of relapse, CAM should be the first choice for treatment in patients with COP who exhibit respiratory sufficiency. Steroids should be considered for patients with a severe and aggressive disease course, particularly patients with underlying conditions (e.g., connective tissue disease, malignancy, or suspected drug reaction). Among patients who fail to respond to steroids, more aggressive treatment with cyclophosphamide, azathioprine, or rituximab should be considered.

## Author contributions

RE and FJ equally contributed to the conception and design of the work and drafted the article. All authors contributed to the article and approved the submitted version.

## Conflict of interest

The authors declare that the research was conducted in the absence of any commercial or financial relationships that could be construed as a potential conflict of interest.

## Publisher’s note

All claims expressed in this article are solely those of the authors and do not necessarily represent those of their affiliated organizations, or those of the publisher, the editors and the reviewers. Any product that may be evaluated in this article, or claim that may be made by its manufacturer, is not guaranteed or endorsed by the publisher.

## Abbreviations

AFOP: acute fibrinous organizing pneumonia
CAM: clarithromycin
CEP: chronic eosinophilic pneumonia
CIOP: cicatricial variant of organizing pneumonia
COP: cryptogenic organizing pneumonia
CT: computed tomography
HRCT: high resolution computed tomography
NSIP: non-specific interstitial pneumonia
OP: organizing pneumonia
PD1: programmed death receptor 1
PD-L1: programmed cell death-ligand 1
PRE: prednisone
ROP: radiotherapy induced organizing pneumonia
SOP: secondary organizing pneumonia
UIP: usual interstitial pneumonia
